# Antipsychotic use in under 25’s - think carefully!

**DOI:** 10.1192/j.eurpsy.2024.928

**Published:** 2024-08-27

**Authors:** D. Collins, R. Holdsworth, T. Nebunu, J. Beezhold

**Affiliations:** ^1^CYFP, NSFT, Great Yarmouth; ^2^ NHS; ^3^Pharmacy, NSFT, Norwich, United Kingdom

## Abstract

**Introduction:**

Antipsychotic use for adolescents (defined here as under 25 year olds) must be done with caution, giving due thought to advantages and potential side effects. Antipsychotics are extremely useful and effective drugs, but have side effects and many of these are problematic.

It has been noted that Risperidone is often used for this age group, despite the UK guidance being cautious about its use.

**Objectives:**

To assess the extent of Risperidone prescribing in Norfolk/Suffolk for this patient group and to consider the monitoring of this.

Given that bone mass density is set down in teens – mid 20’s, this is a particularly concerning issue when given to this age group. Additionally, distressing side effects and issues with fertility shoudl be considered. If risperidone is used, Maudsley is very clear that this must be monitored: baseline/annual prolactin levels done, and action should be taken if these are elevated and/or the patient symptomatic.

**Methods:**

1. Evaluate numbers of adolescents, under 2ndry care Mental health service who have been prescribed Risperidone
2. Consider who prescibed it and the indication
3. to consider if routine monitoring had been completed (specifically, baseline prolactin and then annual prolactin levels)
4. to consider if these patients had developed side effects

**Results:**

Almost 20% of 18-25 years olds, due to be seen in Youth Community Service had been prescribed Risperidone. Of these, only 44% had had prolactin levels done, despite the guidance. This equates to the over half not having prolactin checked. 60% of patients reveiwed had symptoms of hyperprolactinemia. Indications for use included emotional dysregulation/EUPD, psychosis, ADHD, OCD/ASD and depression

**Conclusions:**

Risperidone should be used with extreme caution in this patient group. Medication can be very useful for some young people experiencing distressing symptoms but, as Hippocrates advises, “do no harm” and seek not to cause iatrogenic harm.

Given that many of the young people seen by mental health services are experiencing emotional dysregulation (not necessarily an abnormal state in adolescent, when much is in flux), it is tempting to consider medication as one means of trying to alleviate distress. There is no clear treatment for dysregulated feelings, and most would accept that psychological support is more appropriate.

**Disclosure of Interest:**

None Declared

